# Investigation of the Flexural and Tensile Properties of Hybrid Polyester Composites Reinforced with Bamboo Fibers and Red Mud Waste

**DOI:** 10.3390/polym17081060

**Published:** 2025-04-15

**Authors:** Alessandro José Gomes dos Santos, Maurício Maia Ribeiro, Alessandro de Castro Corrêa, Jean da Silva Rodrigues, Douglas Santos Silva, Raí Felipe Pereira Junio, Sergio Neves Monteiro

**Affiliations:** 1 Department of Industrial Engineering, Federal University of Pará—UFPA, Ramal Manoel de Abreu, S/Nº, Mutirão, Abaetetuba 68440-000, PA, Brazil; ajgs@ufpa.br; 2 Federal Institute of Education, Science and Technology of Pará—IFPA, Estrada do Icuí Guajará, Ananindeua 67125-000, PA, Brazil; mauricio.maia@ifpa.edu.br; 3 Materials Engineering Program, Federal Institute of Education, Science and Technology of Pará—IFPA, Avenida Almirante Barroso, 1155, Marco, Belém 66093-020, PA, Brazil; alessandro.correa@ifpa.edu.br (A.d.C.C.); jean.rodrigues@ifpa.edu.br (J.d.S.R.); 4 Department of Materials Science, Military Institute of Engineering—IME, Praça General Tibúrcio, 80, Praia Vermelha, Urca, Rio de Janeiro 22290-270, RJ, Brazil; raivsjfelipe@ime.eb.br (R.F.P.J.); sergio.neves@ime.eb.br (S.N.M.)

**Keywords:** hybrid composites, polyester, bamboo fibers, red mud waste

## Abstract

This article discusses research on utilizing natural fibers and red mud waste as eco-friendly alternatives in the production of polymer matrix composites. In this study, composites of isophthalic unsaturated polyester matrix were produced by combining bamboo fibers (*Bambusa vulgaris*) and red mud waste. The red mud waste utilized had a particle size of 50–100 mesh, and the fibers measured 15 mm and 30 mm in length, distributed randomly throughout the matrix. Bamboo fibers were utilized in their raw form and underwent treatment with NaOH (5% for 2 h). The composites underwent mechanical assessment via flexural and tensile testing. The mechanical properties measured were analyzed using analysis of variance (ANOVA) and Tukey’s test. The fracture surfaces of the composites were examined using Scanning Electron Microscopy (SEM). Composites featuring 30 mm long treated fibers and 30% red mud exhibited improved flexural strength (124.71 MPa), along with a deformation of 2.16 mm and a flexural modulus of 15.79 GPa. Tensile tests revealed that incorporating red mud waste significantly enhanced the tensile strength by 68% (15BTRMW10) compared to neat polyester. ANOVA confirmed the dependability of the findings, emphasizing the viability of producing hybrid composites from red mud waste and bamboo fiber.

## 1. Introduction

The growing demand for environmentally responsible solutions has accelerated the replacement of conventional materials with sustainable, low-impact, and cost-effective alternatives. This trend is driven by several factors, including the accumulation of industrial waste, the intensifying effects of climate change, the progressive depletion of fossil resources, and the rising cost of petroleum-based products. In this context, the development of new materials that combine technical performance with ecological responsibility has become essential, particularly in industrial sectors that require innovation aligned with sustainability [1,2].

As a response to these demands, natural-fiber-reinforced composites (NFRCs) have emerged as promising alternatives to traditional synthetic composites. These materials offer significant advantages, such as biodegradability, renewability, low density, high specific strength, and lower processing energy requirements [3,4]. In recent decades, the use of natural-fiber-reinforced composites has expanded considerably in the field of materials engineering, particularly in structural and semi-structural applications, enabling the partial or even total replacement of conventional materials like steel, aluminum, and glass fiber in specific use cases [5,6].

Polymeric matrix composites, especially those combined with natural fibers or particulate fillers, exhibit adjustable mechanical, thermal, chemical, and barrier properties. Structurally, these composites consist of a polymeric matrix (either thermoset or thermoplastic) that serves as the continuous phase, which is responsible for encapsulating and transferring load to the reinforcement, and a dispersed phase made of fibers or particulates that impart strength, stiffness, and dimensional stability [7,8]. The incorporation of plant-based fibers and industrial solid waste, such as red mud, not only enhances the functional properties of the composite but also adds value to underutilized materials, reduces production costs, and reinforces commitment to sustainable practices. This hybrid approach represents an innovative strategy for developing high-performance and environmentally viable composite materials, aligning with circular economy principles [7,8,9].

**Figure 1:**
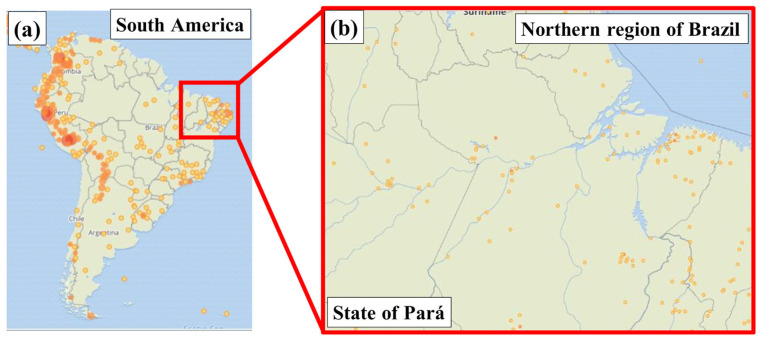
Locations where the species *Bambusa vulgaris* is present. (**a**) South America and (**b**) state of Pará (northern region of Brazil).

Bamboo is considered one of the most appealing biofibers due to its numerous advantages, including its low environmental impact, renewability, fast growth, and comparatively high strength when set against other natural fibers like jute and cotton [10]. Asian nations like China and India account for more than 80% of the global supply of bamboo fiber [11]. Bamboo fibers have been utilized as reinforcement in polymer composites, and numerous studies have shown the potential of bamboo fiber reinforcement across various polymer composites [7]. According to the Gbif website (2025), which reports the occurrence of several plant species in the world, *Bambusa vulgaris* is present in several countries, with incidence also in Brazil, as shown in Figure 1.

In industries that produce alumina, red mud is created as a waste by-product during the Bayer process. To create one ton of alumina, as much as two tons of red mud is produced. These firm blends pose environmental risks due to their highly alkaline properties. Around 120 Mt of red mud is said to be generated each year globally [12].

Hybrid composites are substances that incorporate two or more reinforcement materials within one matrix. The reinforcement can consist of two distinct fibers, two separate filler particles, or fibers combined with filler particles. These fillers and particle reinforcements in the matrix provide outstanding mechanical properties. Hybrid composites that incorporate industrial waste and natural fiber reinforcement present two key benefits. Firstly, they can minimize and repurpose industrial waste effectively; secondly, the use of natural fiber reinforcements allows for the attainment of strong mechanical properties [13].

Chen et al. [14] created hybrid composites of red mud reinforced with waste paper utilizing a polypropylene matrix. The proportion of red mud was altered to 30, 40, and 50 wt%. The hybrid composite demonstrated better bending strength compared to the neat PP. Waste paper and red mud mixed in a 6:4 ratio exhibited a peak bending strength of 50 MPa and a tensile strength of 19 MPa. Nonetheless, the hybrid composite of red mud and waste paper created at a 5:5 ratio showed low tensile (16 MPa) and flexural strength (48 MPa), which the authors attributed to agglomeration. The clustering of the red mud particles heightened stress concentration, resulting in failure. All of these studies have demonstrated that agglomeration poses a significant issue when utilizing red mud as reinforcement. Discovering a method to lower the buildup of red mud might enable enhanced strength at a greater loading percentage of red mud.

Akinci et al. [15] manufactured composites of isotactic polypropylene polymer matrix by utilizing red mud as the filler. The analysis of mechanical properties indicated that as the red mud content increased, both tensile strength and flexural strength displayed a linear decreasing trend. Nevertheless, the hardness of the composites rose as the red mud content increased.

Gandhi et al. [16] infused a modified *Cissus quadrangularis* stem fiber-reinforced epoxy composite with red mud. The researchers discovered that incorporating a suitable quantity of red mud enhanced the composite’s mechanical properties.

Arumugaprabu et al. [17] examined the influence of varying quantities of red mud on polyester/banana fiber composites. The red mud dimensions were 4, 6, and 13 μm, with reinforcement percentages of 2, 4, 6, 8, and 10 wt%. Reinforcement was provided by 30 mm short banana fiber, with the fiber reinforcement percentage kept at 30 wt%. The reinforcement of smaller red mud particles was discovered to enhance the composite’s strength. The hybrid red mud/banana fiber composite exhibited greater tensile strength at 8 wt% reinforcement with 4 μm sized red mud, which was 50% more than the composite lacking red mud.

Prabu et al. [18] incorporated and altered polyester and epoxy resins with red mud and examined their mechanical properties. They discovered that the tensile and impact strengths of red mud–polyester resin/epoxy resin composites exhibited decreasing trends, while the hardness of the composites rose.

Vigneshwaran et al. [19] discussed the impact of different fiber loading levels in polyester composites made of red mud and sisal fiber. The percentage of fiber weight was adjusted to 20, 30, and 40 wt%, while the weight percentage of red mud was altered to 10, 20, and 30 wt%, with an average particle size of 0.7 μm. Hybrid composites demonstrated changes in hardness, tensile, and flexural characteristics based on fiber loading. The highest hardness was observed for a composite with 20 wt% fiber and 30 wt% red mud, the greatest tensile strength for a composite with 40 wt% fiber and 20 wt% red mud, and the highest flexural strength for a composite with 30 wt% fiber and 20 wt% red mud. This variation highlights the significance of determining an ideal fiber weight percentage in the production of red mud hybrid composites.

Biswas et al. [20] investigated how red mud filler influences the mechanical properties of bamboo-fiber-reinforced epoxy composites. It was observed that the tensile and flexural strength of bamboo-fiber-reinforced epoxy composites significantly improved as the red mud content increased (tensile strength increased from 10.5 MPa to 12.5 MPa; flexural strength increased from 20 MPa to 28 MPa); however, the hardness and impact strength diminished (hardness from 46 Hv to 33 Hv, impact strength from 1.4 J to 0.1 J).

In a different investigation, Arumugaprabu et al. [21] evaluated the strength of the hybrid red mud/banana polyester composite against the red mud/sisal polyester composite. Both fibers were reinforced as short fibers (10 mm), maintaining a constant weight percentage of fiber (20 wt%) and red mud (10 wt%). The sisal/red mud composite exhibited greater tensile strength (35 MPa), flexural strength (approximately 90 MPa), and impact resistance (approximately 11 J) compared to the banana/red mud composite. The writer advised utilizing red mud/sisal fiber composites for applications that require load bearing.

Zhang et al. [22] produced red mud/polypropylene (PP) composites utilizing twin-screw extruders and additional machinery. Examination of their mechanical characteristics revealed that at 15% red mud content, the composite attained its peak tensile strength. Nonetheless, as the red mud content rose, the performance characteristics of the composites diminished. Consequently, red mud/PP composites demonstrated improved mechanical properties with relatively low red mud levels.

Despite recent advances in the development of composites reinforced with natural fibers, there is still a significant lack of data on the mechanical properties of hybrid systems composed of polyester, bamboo fibers, and industrial waste, such as red mud. This knowledge gap reinforces the importance of studies that explore the structural and functional behavior of these materials, especially studies targeting engineering applications that require satisfactory mechanical performance. The search for sustainable materials with properties equivalent or superior to those of traditional synthetic composites, such as those reinforced with glass fibers, is one of the main drivers of this research. In this context, the present investigation contributes significantly to the literature by providing unprecedented and systematized data on an innovative hybrid composite, scientifically validating its applicability through several physical–mechanical characterizations.

The originality of this work lies in the formulation and evaluation of hybrid polymer composites that incorporate bamboo fibers, both in their natural state and chemically treated, associated with different levels of red mud residues—an industrial by-product with potential for reuse. This approach promotes not only the valorization of renewable resources and solid waste but also results in the production of materials with technical properties adapted to the materials processing sector. Furthermore, the composites were subjected to a wide range of tests, including mechanical, morphological, and statistical analyses, allowing for an in-depth understanding of their performance.

Another strategic differentiator of the project is its alignment with the principles of the circular economy and environmental sustainability. The incorporation of industrial by-products that are traditionally discarded into high-value-added formulations represents a promising approach from both an academic and industrial perspective. Thus, the results obtained not only contribute to the advancement of technical–scientific knowledge but also offer viable technological solutions for sectors seeking ecological alternatives to conventional materials.

## 2. Materials and Methods

### 2.1. Materials

**Figure 2:**
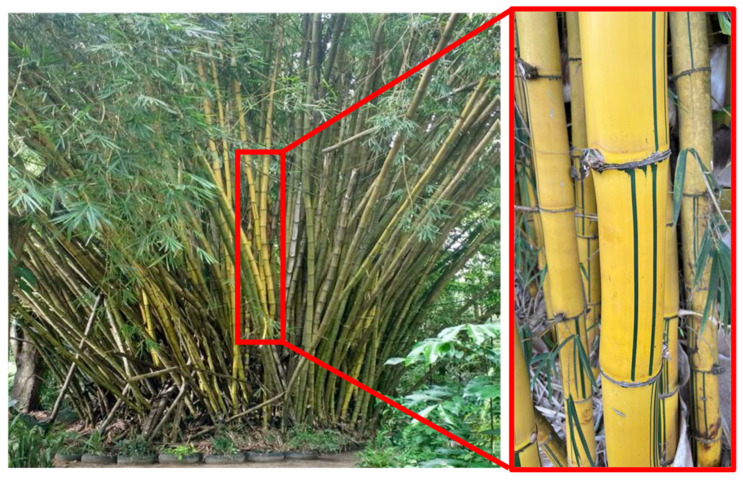
Photograph of bamboo (*Bambusa vulgaris*), with a focus on the stalks.

**Figure 3:**
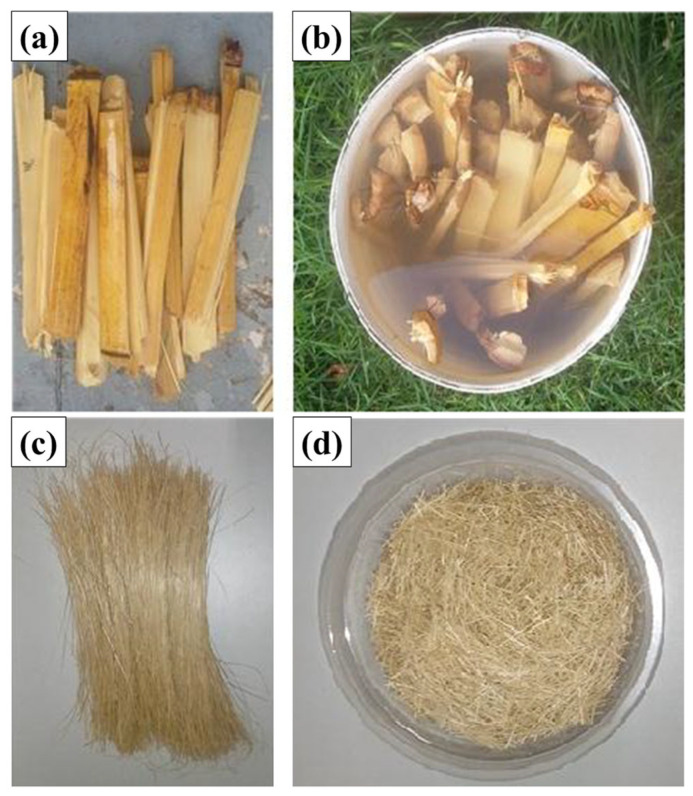
Stages of obtaining fibers: (**a**) sliced stalks, (**b**) submerged stalks, (**c**) extracted fibers, and (**d**) fibers cut into 15 mm lengths.

The medium viscosity unsaturated terephthalate-based polyester resin (Arazyn AZ 1.0 #34) and the methyl-ethyl-ketone peroxide (MEK) catalyst, PERMEC D-45, provided by Ara Química SA (São Paulo, Brazil) were utilized as the hybrid composite polymer matrix. The polyester resin was combined with 1.0 wt% of a hardener. Bamboo fibers (*Bambusa vulgaris*), obtained from a bamboo stalk in the forest at the Campus of the Federal University of Pará (UFPA), served as reinforcement (Figure 2). The bamboo stalks were chopped, after which they underwent the maceration process, where they were “immersed” for a set duration of 7 days, aiming to detach the fibers from the woody section of the stalk (Figure 3). Following the maceration phase, the fibers were placed in the circulation oven (model MA 035/5, MARCONI brand, Algodoal, Brazil) for 30 min at a temperature of 105 °C for 5 min to eliminate the moisture present in the fiber itself. Subsequently, the bamboo underwent the defibrillation procedure.

#### Red Mud Waste

Red mud is an insoluble residue generated in the process of obtaining aluminum from bauxite through the Bayer process, which is disposed of in suitable locations called disposal ponds. Red mud has a high alkaline content and is composed of fine particles of oxides and hydroxides of silica, aluminum, iron, calcium, and titanium, and it may also contain trace elements of oxides of other metals [23].

It is noted that there is growing concern regarding the development new heavy metal adsorbents using available materials, such as industrial waste. Studies have shown the use of fly ash, blast furnace sludge, sludge, tea factory waste, and beet pulp, among others, as low-cost adsorbents for heavy metals in wastewater, in addition to red mud, one of the most promising adsorbents currently available [24].

Regarding the properties of red mud, these vary significantly between different bauxites and different production methods; however, the basic properties are high pH (ranging from 10 to 12.5) and extremely fine distribution of suspended solids with a content of 15 to 30%. This waste has a complex chemical composition, and, due to the high levels of calcium and sodium hydroxide in association with the large quantities produced annually, it is relatively toxic and a serious environmental pollutant [23].

**Figure 4:**
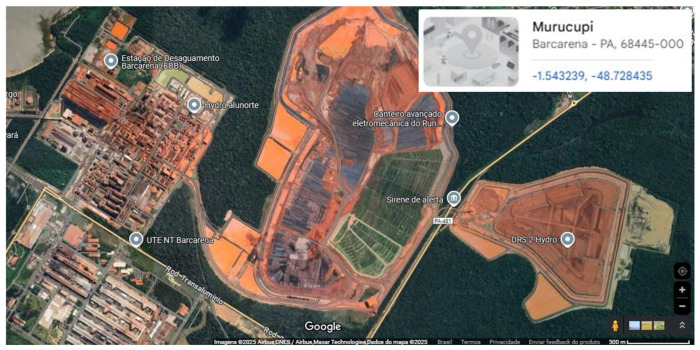
Aerial photograph of the Hydro Alunorte plant located in Barcarena, northern Brazil. Source: author, via Google Earth (March 2025).

The storage of mud, which was previously performed using storage ponds, has been replaced by the dry stacking method, which is more efficient and safer for the environment, a method that has already been adopted by Hydro Alunorte. This industrial plant shows a superficial image of the factory area and waste pond, as seen in Figure 4.

### 2.2. Alkaline Treatment of Bamboo Fibers

Bamboo fibers were subjected to an alkaline treatment with the aim of improving interfacial adhesion with the polymer matrix and reducing the presence of surface impurities. For this purpose, the fibers were initially washed with distilled water and dried in an oven at 60 °C for 24 h. They were then immersed in an aqueous solution of sodium hydroxide (NaOH) at 5% by mass for a period of 2 h at room temperature (~25 °C). After treatment, the fibers were carefully washed with distilled water until they reached neutral pH and dried again in an oven at 60 °C for 24 h. This process aimed to partially remove hemicelluloses, amorphous lignin, and other non-cellulosic substances, promoting a rougher and chemically active surface. The effectiveness of the treatment was subsequently verified through Scanning Electron Microscopy (SEM) analysis by comparing the mechanical performance of composites with treated and untreated fibers.

### 2.3. Density Determination of Red Mud Waste

The assessment of red mud waste’s density adhered to the protocols of NBR NM 52/2009 [25], employing a pycnometric method with distilled water and three samples of about 1.0 g of particulates.

### 2.4. Processing of Bamboo/Red Mud Composites

The bamboo/red mud hybrid composites (BRMHC) were produced using the compression molding technique by employing a stainless-steel mold measuring 300 × 160 × 2.5 mm. In the mold, 3 wt% of bamboo fibers with random orientations was incorporated. Sample groups were created based on variations in fiber length, consisting of groups with fibers measuring 15 mm and 30 mm long. Before processing, the fibers were treated with mercerization using a 0.05 NaOH solution for 2 h.

**Figure 5:**
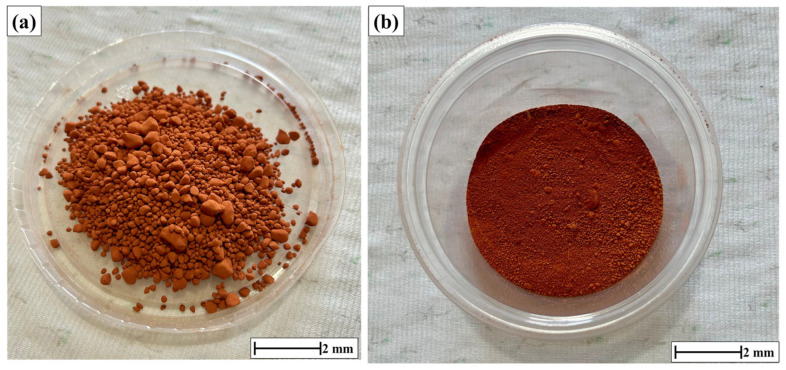
(**a**) Red mud waste as acquired and (**b**) red mud waste dimensions of 50–100 mesh.

The red mud waste was subjected to drying in an oven at 100 °C for 24 h. Once dried, the red mud underwent sieving to achieve a particle size of 50–100 mesh to obtain the red mud utilized in the current study, as shown in Figure 5.

**Figure 6:**
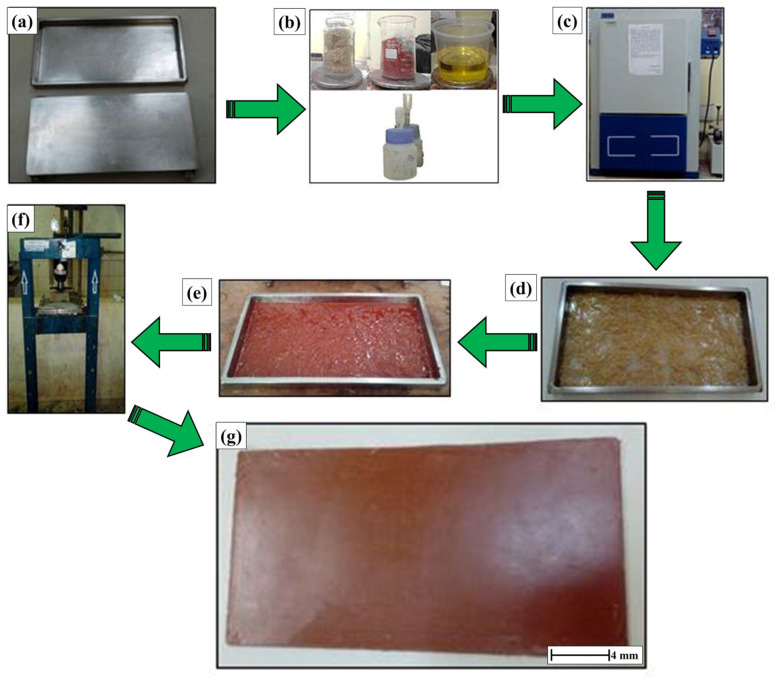
Composite manufacturing steps. (**a**) Metal mold after application of the release agent, (**b**) determination of the masses of the fibers, residue (red mud), resin, and volumes of the cobalt accelerator and catalyst, (**c**) oven used to remove moisture from the fiber and residue, (**d**) fibers randomly distributed in the metal mold, (**e**) initial curing process, (**f**) pressing of the material in the hydraulic press, and (**g**) plates after the curing process.

Subsequently, the red mud powder was combined with polyester resin through mechanical stirring, continuing under low-speed mixing for 10 min, during which the mixture’s uniformity was monitored. The red mud was incorporated into the resin in three varying amounts: 10, 20, and 30 wt%. Following homogenization, 1 wt% of MEK catalyst was introduced to the blend. Subsequently, the blend was transferred into the steel mold containing bamboo fibers. To prepare the composites, the plates were fabricated in a metal mold, and the gel time (the material during curing) was found to be between 15 and 20 min. Right after that, the metal mold was shut and pressed in a hydraulic press (model MPH-15, MARCON brand, Marília, Brazil) applying a force of 2.5 kN for 20 min. The plates were taken out of the metal molds and allowed to sit at room temperature for the entire curing procedure, which lasted 24 h. Following the complete curing process, the composite plates were sliced on a circular bench saw to create the test specimens as per the standards for each examination. The visual examination conducted on the produced composites showed uniform materials with minimal air bubble presence linked to processing mistakes. This accomplishment might have been possible due to how the load was introduced during hydraulic press processing; it was applied slowly and gradually, allowing the air trapped between the fibers/red mud waste in the material ample time to escape. Figure 6 shows the composite manufacturing process.

Table 1 displays the nomenclature and makeup of the examined groups, where the categorization of each group is determined by the red mud content, dimensions, and treatment of the bamboo fibers.

### 2.5. Density Determination of Composites

Density measurements were conducted on composites measuring 25 × 25 mm based on the Archimedean principle. The cured composite was subsequently weighed in air and then measured again in a liquid with a known density. The density obtained from the measured values was presented in g/cm^3^, as illustrated in Equation (1).

(1) $$D=\frac{\left(M_{s}\times \rho_{L}\right)}{\left(M_{U}-M_{I}\right)}(g/{\mathrm{cm}}^{3})$$

In this context, *M_U_* represents the wet mass (g), *M_S_* denotes the dry mass (g), *M_I_* refers to the immersed mass (g), and ρL indicates the specific mass of water (g/cm^3^).

### 2.6. Flexural Tests

The hybrid composites’ flexural strength, deflection, and flexural modulus were assessed using a three-point bending test as per ASTM D 790 [26]. A testing machine, EMIC/DL500, fitted with a 5 kN load cell was utilized. Flexural tests were performed on eight specimens under each condition, amounting to a total of 104 specimens, with a span of 80 mm, a testing speed of 2.5 mm/min, and a pre-load of 3 N. Throughout the test, every specimen broke within the standard length. Utilizing the stress–strain curves, the flexural strength (*σ*) and flexural modulus (*E*) of the composite were determined in accordance with Equation (2).

(2) $$\sigma =\frac{3PL}{2BW^{2}}$$

where *P* denotes the peak load achieved, *L* indicates the distance between supports, and *B* and *W* represent the width and thickness of the samples, respectively. The flexural modulus (*E*) is calculated using Equation (3).

(3) $$E=\frac{mL^{3}}{4BW^{3}}$$

where *m* represents the slope of the load versus the deflection curve.

### 2.7. Tensile Tests

The tensile tests were performed on 8 specimens for each condition, amounting to a total of 104 specimens, at 23 °C in accordance with the ASTM D3039 [27] standard, utilizing a KRATOS model IKCL3 universal testing machine equipped with a 5 kN load cell. The crosshead movement was established at 2 mm/min for every tensile test.

### 2.8. Statistical Analysis

The analysis of variance (ANOVA) was conducted to assess the statistical significance of the primary effects of factors and the interaction among factors in the responses. To evaluate the means of treatments, Tukey’s Significant Difference Test (post hoc test) was utilized. The Shapiro–Wilk and Levene tests were utilized to assess the ANOVA assumptions, including, specifically, the normal distribution of residuals and the equality of variances.

### 2.9. Scanning Electron Microscopy (SEM)

The composite fracture surfaces were examined with a Hitachi scanning electron microscope, model TM3000. The voltage of the electron beam was adjusted to 20 kV. The specimens were covered with a thin layer of gold through a cathodic sputter model ACE600 (Leica, Wetzlar, Germany) for 30 min.

## 3. Results and Discussion

### 3.1. Density Analysis of Composites

Table 2 shows the density results for the hybrid composites.

The density results indicate the combined effect of red mud and bamboo fibers on the polyester matrix, showing that the addition of inorganic and organic reinforcements increases the density in relation to pure polyester (1.20 ± 0.09 g/cm^3^). In general, increasing the red mud content (10%, 20% and 30%) promotes progressive increases in density, as it is a mineral load denser than the matrix. The density value found for red mud was 2.04 g/cm^3^. In composites with 30% red mud, values close to 1.50–1.54 g/cm^3^ are observed, well above the 1.20 g/cm^3^ of neat polyester and formulations with 10% red mud (around 1.24–1.27 g/cm^3^).

The presence of bamboo fibers also influences the density, although to a lesser extent than red mud. When comparing the same fraction of red mud, the length of the fibers (15 mm or 30 mm) and the chemical treatment (untreated or treated) can slightly alter the values. In general, treated fibers tend to have slightly higher densities than untreated fibers, possibly due to the removal of waxes and impurities, which improves adhesion and may decrease internal porosity. Thus, it is observed that the formulations 15BTRMW10, 30BTRMW10, and 30BTRMW20, for example, present slightly higher densities than the untreated versions with the same fraction of red mud and fibers.

The length of the fibers (15 mm versus 30 mm) also has an influence, albeit in a subtle way. In some cases, changing to longer fibers results in an increase in the final density, as longer fibers can favor less void formation, as long as they are well-impregnated by the matrix. On the other hand, if the mixing and compaction process is not adequate, longer fibers can generate heterogeneous dispersion, influencing the variation of values (increasing the standard deviation).

In general, red mud is the main factor in increasing density, as its increasing content from 10% to 30% results in the most significant jumps. Bamboo fibers, whether treated or not, contribute in a secondary way, being able to adjust the final density and introduce small variations depending on the length and chemical treatment. The standard deviations indicate good repeatability in most formulations, although some, especially with 30% red mud, exhibit greater dispersion, possibly associated with the difficulty of homogenizing the composite when high levels of mineral filler and fibers accumulate.

### 3.2. Flexural Properties of Composites

The findings regarding flexural strength, deflection, and the flexural modulus of hybrid composites are shown in Table 3.

Table 3 presents twelve composite conditions, in addition to neat polyester, varying the red mud residue content (10%, 20%, and 30%), the bamboo fiber length (15 mm or 30 mm), and the chemical treatment (untreated or treated fiber). Each condition was evaluated in terms of flexural strength limit (MPa), deflection (mm), and flexural modulus (GPa), with uncertainties (standard deviations) indicating statistically significant differences when the variation between the means exceeds the sum of these deviations.

Neat polyester serves as a reference. Its flexural strength, around 82–83 MPa, tends to be lower than that of reinforced composites, and its flexural modulus, around 13–14 GPa, is also lower than that of most fiber and red mud formulations. On the other hand, NP usually presents greater deflection than composites, as it does not contain reinforcements that increase rigidity. Thus, compared to the other conditions, NP generally exhibits the worst performance in strength and modulus, but it is not the worst in deflection capacity, as it deforms more before fracturing.

In composites with untreated fiber, the addition of 10% to 30% red mud increases the flexural strength above the NP value while increasing the modulus. This is because bamboo fiber, even without treatment, provides additional tensile strength, while red mud acts as a mineral filler, making the material more rigid. However, deflection tends to decrease as the matrix becomes less ductile. In formulations with 30% red mud, if there are agglomerations or inadequate dispersion, the gain in strength may not be as significant as expected, and it may be statistically close to values of 20%. Longer fibers (30 mm) typically improve strength and modulus even further as there is more load transfer surface, although this further reduces deflection.

In composites with treated fiber, the removal of lignin and impurities improves matrix–fiber adhesion, resulting in additional and statistically significant gains in strength and modulus compared to untreated composites. In these cases, it is common for the flexural strength limit to exceed the range of 100–110 MPa or more, depending on the combination of fiber length and red mud content. The modulus also increases, often exceeding 15 GPa in the best formulations. On the other hand, the deflection tends to be even lower, as the more effective fiber–matrix interface causes the composite to rupture at higher stresses but with less elongation.

When comparing extreme conditions, the worst performance in strength and modulus is generally that of NP, which, despite having greater deflection, does not reach significant stress values before fracturing. The best resistances and modules are usually obtained in composites that combine treated fiber (30 mm) and intermediate or high levels of red mud (20% or 30%). In these formulations, it is observed that the flexural strength limit and the modulus are statistically higher, although the deflection remains low due to the rigidity of the system. If the red mud content is very high (30%) and there are dispersion problems, there may be a small drop or results statistically close to 20%. Still, in general terms, these combinations (treated fiber, longer length, and moderate to high residue content) provide the best balance between high strength and high flexural modulus, at the cost of lower deflection.

In short, the analysis confirms that neat polyester has the worst performance in terms of flexural strength and modulus, while composites with a higher content of red mud and bamboo fibers (especially treated and 30 mm) present higher strength and modulus values, but with lower deflection. Thus, each variable—chemical treatment, fiber length, and residue content—plays a crucial role in improving bending behavior, but it is necessary to balance stiffness and strength with the inevitable decrease in deformation capacity.

Going further, in relation to flexural strength, the results showed that intermediate levels of red mud, especially 20%, tend to promote superior mechanical performance in most formulations with untreated fibers. For example, the 15BURMW20 composite achieved 115.51 ± 5.96 MPa, outperforming its 10% and 30% counterparts. However, when the bamboo fibers were treated with NaOH and combined with a longer length (30 mm), the composites showed more consistent and higher performance even with 30% red mud. This was the case for the 30BTRMW30 composite, which obtained the highest flexural strength value among all of the groups tested, with 124.71 ± 4.35 MPa.

Maximum deflection, which indicates the ductility of the composites, also showed a pattern related to the red mud content. In many cases, increasing the red mud content up to 20% resulted in greater deformation before failure. However, this behavior was not maintained at 30% for all groups. Some composites showed a slight decrease in deflection, suggesting that particle overload may compromise matrix mobility. Notably, the 30BTRMW30 composite again stood out, with a deflection of 2.16 ± 0.33 mm, indicating that with the correct formulation, even high red mud contents do not compromise deformation capacity.

The flexural modulus, in turn, showed a progressive increase with the increase in the red mud content, especially in composites with treated fibers. The presence of the residue contributed to the increase in the rigidity of the materials, functioning as a structural ceramic filler. The highest value was also observed for 30BTRMW30, which reached 15.79 ± 1.13 GPa. This indicates that under conditions of good interfacial compatibility and adequate dispersion, the red mud acts efficiently as reinforcement.

It can be seen that increasing the red mud content favors gains in rigidity and, to a certain extent, also in resistance, although it may compromise ductility in some cases. The quality of the residue dispersion and its compatibility with the polymer matrix and fibers are determining factors for the success of the formulation. Thus, the combination of chemically treated fibers, adequate length, and optimized red mud contents proved to be essential for obtaining composites with superior mechanical properties.

Although the bamboo fiber content was kept constant at 3% by mass in all formulations in the study, a significant variation was observed in the mechanical properties of the composites. This indicates that in addition to the mass fraction, other factors related to the morphology and surface condition of the fibers play a fundamental role in the performance of the materials. Among the factors evaluated, the fiber length, the alkaline treatment, and the interaction with the red mud residue content stand out.

The variation in fiber length, between 15 mm and 30 mm, directly impacted the strength and stiffness of the composites. Longer fibers increase the interface area with the matrix, favoring better load transfer, in addition to contributing to the formation of a more intertwined internal structure, which improves stress distribution and slows crack propagation. This is evidenced by the superior performance of sample 30BTRMW30 (30 mm fiber, treated, 30% red mud), which achieved a flexural strength of 124.71 ± 4.35 MPa and a flexural modulus of 15.79 ± 1.13 GPa, the highest values among all of the composites evaluated.

Furthermore, alkaline treatment of the fibers with a 5% NaOH solution for 2 h also proved effective in improving fiber–matrix interfacial adhesion. This treatment promotes the removal of surface impurities and hemicelluloses, increasing the surface roughness of the fibers and favoring mechanical anchoring. Thus, it was possible to observe that composites with treated fibers presented, in general, better mechanical performance compared to those produced with raw fibers.

Another factor that influenced the mechanical behavior was the content of red mud residue incorporated into the matrix. Although this material functions as a mineral filler, contributing to the increase in the rigidity of the system, its interaction with the fibrous structure appears to enhance the reinforcement effect, especially when associated with long and treated fibers. The 30BTRMW30 composite once again stands out in this context, evidencing positive synergy between the constituents.

In other words, even with the fiber content kept fixed, the fiber length, surface chemical treatment, and mineral filler content significantly influence the mechanical properties of hybrid composites. These results reinforce the importance of the microstructural design of composite materials, going beyond simple variation in the reinforcement proportion.

### 3.3. Tensile Properties of Composites

Table 4 displays the outcomes of tensile strength, total strain, and Young’s modulus for hybrid composites.

Table 4 compares thirteen conditions in total, including neat polyester (NP) and twelve more composites obtained by varying the red mud residue content (10%, 20%, and 30%), the length of the bamboo fiber (15 mm or 30 mm), and the chemical treatment (untreated or treated fiber). The results include the tensile strength limit (MPa), deformation (mm/mm), and the modulus of elasticity (GPa), with standard deviations that allow for the identification of statistically significant differences when the difference between the means exceeds the sum of these deviations.

Neat polyester (NP) serves as a reference and has the lowest strength (around 23 MPa), the lowest modulus (around 0.92 GPa) and the highest deformation (around 0.0316 mm/mm). Any addition of fibers and waste tends to improve the strength and modulus, although it reduces the deformation.

In composites with untreated fiber, the presence of red mud (10%, 20%, or 30%) and bamboo (15 mm or 30 mm) increases the strength and modulus in relation to NP, as the fiber adds tensile capacity and the mineral residue increases rigidity. However, deformation falls as the matrix becomes less ductile. If the red mud content reaches 30% without good dispersion, there may be specific failures that limit the gain in strength. Furthermore, 30 mm fibers typically provide higher strength and modulus than 15 mm fibers because they offer a larger load transfer area, although this further reduces deformation.

In composites with treated fiber, chemical treatment (e.g., alkaline) removes waxes and part of the lignin, improving fiber–matrix adhesion. In general, these composites have statistically superior strength and modulus compared to their untreated counterparts, especially when combining 20% or 30% red mud and 30 mm fibers. Under these conditions, the strength typically exceeds 30 MPa, and the modulus can approach or exceed 1.1 to 1.2 GPa depending on the dispersion of the residue. On the other hand, deformation tends to be even lower, as the material becomes stiffer and breaks at higher stresses but with little elongation.

Comparing the extremes in each parameter, the worst performance in strength and modulus occurs with NP, which, on the other hand, has the greatest deformation. The best results in terms of tensile strength and modulus of elasticity tend to appear in composites that combine treated fiber (30 mm) and 20% or 30% red mud, as long as the dispersion is adequate. If the amount of residue is very high and poorly distributed, the performance may equal that of 20%, not exceeding this intermediate content. In terms of deformation, the tendency is for composites with greater rigidity (more red mud, treated, and long fibers) to present the lowest values, as the gain in rigidity sacrifices ductility.

Thus, neat polyester stands out for its ductility, but it exhibits the lowest resistance and the lowest modulus. The introduction of bamboo fibers, even untreated, already improves these aspects, and the red mud further reinforces rigidity and resistance. However, the chemical treatment of the fibers and the use of longer fibers (30 mm) enhance the gains, resulting in composites that reach maximum strength and modulus values, although with much lower deflections than in NP. In this way, each variable—red mud content, fiber treatment, and fiber length—plays a decisive role in optimizing mechanical properties, with the best balance generally arising in composites with treated fibers that are 30 mm long and with 20% red mud or 30% if the dispersion is homogeneous.

Going further, in the case of tensile strength (T.S.), samples with 10% red mud generally presented the best performances compared to those with 20% and 30%. For example, the 15BURMW10 composite reached 36.33 ± 2.44 MPa, a value considerably higher than that obtained by 15BURMW30 (29.23 ± 3.49 MPa). Similar behavior was observed in the samples with treated fibers, in which 15BTRMW10 obtained 32.28 ± 5.18 MPa, surpassing 15BTRMW30 (29.23 ± 3.49 MPa) and, mainly, 30BTRMW30 (24.39 ± 2.91 MPa). These results indicate that red mud, when present in high levels, can act as a weak point within the matrix, reducing the efficiency of load transfer between the matrix and the fibrous reinforcement.

The analysis of total strain (To.S.) reinforces this interpretation. A slight reduction in strain values was observed with increasing red mud content, especially in formulations with 30% filler. This indicates a loss of ductility of the composite, making it stiffer and less capable of elastic deformation before failure. Composite 30BTRMW30, for example, presented a strain of only 0.0328 ± 0.0032 mm/mm, while 15BURMW10 reached 0.0351 ± 0.0043 mm/mm.

Regarding Young’s modulus (Y.M.), which represents the material’s stiffness, the trend was similar; the highest values were observed in composites with 10% red mud, such as 15BURMW10 (1.275 ± 0.233 GPa), with a reduction as the residue content increased, as in the case of 30BURMW30 (0.723 ± 0.055 GPa). This decrease suggests that although red mud is a ceramic waste, its excess does not necessarily contribute to increased mechanical stiffness, especially when there is no good dispersion or compatibility with the matrix.

On the other hand, it is noted that the presence of chemically treated fibers contributed to partially mitigating the negative effects of the increase in mineral load. In several comparisons, composites with treated fibers presented greater stability in strength and modulus values, demonstrating that adequate interfacial adhesion between fibers and the matrix allows for better use of the properties of the constituents, even with a higher mineral particle content.

It can be seen that increasing the red mud content tends to reduce both the strength and stiffness of composites in tension. This reduction is associated with a decrease in the structural cohesion of the matrix and the possible formation of weak zones or discontinuities with excess load. However, the use of treated fibers and the control of fiber length can reduce the negative impacts, maintaining satisfactory mechanical performance even in formulations with high red mud content. The choice of the ideal content should therefore seek a balance between stiffness, strength, and toughness while also considering the synergistic effects between the constituents of the composite.

Table 5 presents a comparison of the results found in the present work with those in the literature.

The comparative analysis between the hybrid composites produced in this study and the results of other authors allows us to clearly observe the competitive performance of the formulations based on polyester, bamboo fibers, and red mud waste both in terms of flexural and tensile strength.

Regarding flexural strength, the 30BTRMW30 composite (30 mm treated fibers and 30% red mud) presented the highest value among all of the listed formulations, with 124.71 ± 4.35 MPa, substantially surpassing the composites of other authors, such as RW-2 (50.19 MPa), PMC-PP-10 (55.4 MPa), and EBR-4 (28.1 MPa). This represents an increase of approximately 149% compared to the RW-2 composite and more than double the strength presented by the EBR-4 formulation, which contains a high fraction of bamboo fibers and red mud (45% + 15%), it but did not reach similar levels of performance. Even when compared to epoxy composites treated with bamboo fibers, such as UETBRMC_15_, which obtained 215.3 MPa of flexural strength, it is important to emphasize that the differences in the polymer matrix (epoxy vs. polyester) and the formulation justify this discrepancy. Considering only composites with a polyester matrix and the inclusion of red mud, the formulations in the present work demonstrate excellent competitiveness.

Regarding tensile strength, the 15BTRMW10 composite (15 mm treated fibers and 10% red mud) obtained the best performance among the samples evaluated in this study, with 38.81 ± 3.84 MPa. This value surpasses all polymer matrix composites with red mud cited in the comparative literature (RW-2 (18.93 MPa), PMC-PP-10 (30.0 MPa), and EBR-4 (12.5 MPa)), demonstrating gains of up to 105% in relation to RW-2 and almost three times more than EBR-4, which presented the lowest value recorded. Even when compared to epoxy systems reinforced with treated bamboo fibers and red mud, such as UETBRMC_15_ (149.2 MPa), which present significantly higher strength, one must consider the fundamental differences in the nature of the matrix. Epoxies, due to their stiffness and adhesion characteristics, tend to present superior tensile strength compared to polyester. Nevertheless, the performance of the 15BTRMW10 composite demonstrates that the combination of polyester, treated fibers, and red mud can achieve tensile strength levels suitable for medium stress engineering applications, with the additional advantage of lower cost and greater sustainability.

Another highlight is the performance of the 15BURMW20 formulation, with untreated fibers and 20% red mud, which reached 115.51 ± 5.96 MPa in flexure and 35.82 ± 3.40 MPa in tension. Even without alkaline treatment, this composite showed superior performance to most formulations in the literature, evidencing the effectiveness of the synergy between intermediate red mud content and adequate fiber length (15 mm).

In other words, the data obtained in this study demonstrate that the correct combination of parameters, such as surface treatment, fiber length, and red mud content, has a significant impact on the mechanical properties of the composites. The developed formulations stand out in relation to several studies in the literature, both in flexure and traction, confirming the technical viability of hybrid composites based on polyester, bamboo fiber, and red mud as a sustainable and efficient alternative for structural and semi-structural applications.

### 3.4. Statistical Analysis

#### 3.4.1. Statistical Analysis of Flexural Properties

The ANOVA results for flexural strength are presented in Table 6, which summarizes the main effects and interactions between the factors. The Shapiro–Wilk (W = 0.979, *p* > 0.05) and Levene [F(11.48) = 0.824, *p* > 0.05)] tests indicated that the ANOVA conditions of normality of residuals and homoscedasticity were satisfied.

It is observed in Table 6 that there were significant main effects for alkali treatment, F(1, 48) = 249.89, *p* < 0.001, indicating that the mean flexural strength was significantly higher in the treated bamboo fiber source (M = 112.11, SD = 14.16) than in the untreated bamboo fiber source (M = 93.91, SD = 12.73) for X2, F(2, 48) = 67.99, *p* < 0.001, and also for fiber length, F(1, 48) = 27.29, *p* < 0.001. There were also significant interactions for X1:X2, which was F(2, 48) = 28.03, *p* < 0.001, X1:X3, which was F(1, 48) = 57.26, *p* < 0.001, X2:X3, which was F(2, 48) = 94.66, *p* < 0.001, and X1:X2:X3, which was F(2, 48) = 7.65, *p* < 0.001.

**Figure 7:**
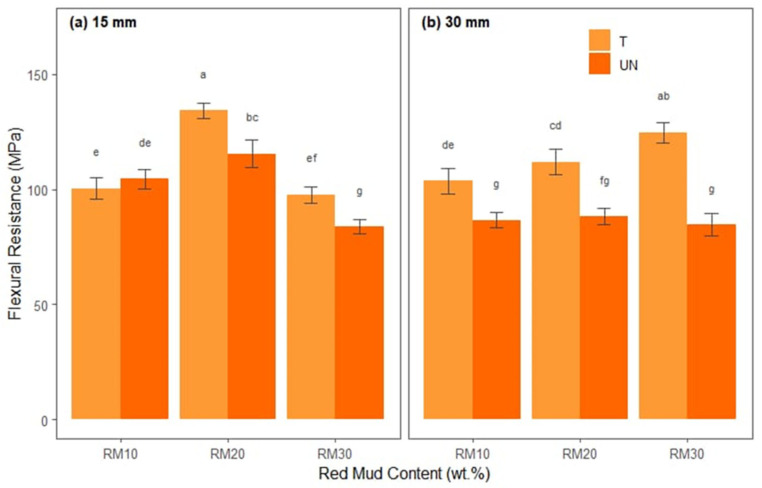
Statistical analysis of flexural strength for hybrid composites: (**a**) reinforced with 15 mm bamboo fibers; (**b**) reinforced with 30 mm bamboo fibers.

Figure 7 shows the average flexural strengths of each treatment combination, separated into fiber lengths (Figure 7a,b), in proportions of red mud residue (*x*-axis) and differentiated by treated and untreated bamboo fibers.

Analysis of the flexural strength graphs (separated into 15 mm and 30 mm fibers with red mud contents of 10%, 20%, and 30%, as well as treated and untreated fibers) reveals some clear trends. In the case of 15 mm fibers (Figure 7a), it can be seen that composites with treated fiber generally achieve higher flexural strength values when compared to untreated versions, indicating that the chemical treatment favors adhesion between the fiber and the matrix, increasing the capacity to withstand flexural stresses. Furthermore, there is a consistent variation in performance as the red mud content increases; some formulations with 20% red mud exhibit significantly higher values (letters “a” or “b” in Tukey’s test), suggesting that this intermediate content provides a good balance between stiffness and toughness. In contrast, increasing the content to 30% may result in local clumping or embrittlement, which for certain combinations reduces strength (e.g., those labeled with letters like “e” or “f”).

For the 30 mm fibers (Figure 7b), the behavior is similar in terms of the influence of the treatment (with emphasis on higher values in the treated composites), but the change in fiber length can alter the dispersion and the form of load transfer, causing some differences in relation to the 15 mm samples. In certain cases, the 30% red mud content, combined with chemical treatment, can still result in high resistance, but if there are problems with impregnation or dispersion, flexure may be impaired (letters “g” or close to it, indicating lower average values). Finally, Tukey’s test revealed that 15BTRMW30 (M = 3.97, SD = 0.59) had a mean deflection that was significantly greater than that of the other treatments.

The ANOVA results for deflection are presented in Table 7, which summarizes the main effects and interactions between the factors. The Shapiro–Wilk (W = 0.985, *p* > 0.05) and Levene [F(11.48) = 1.122, *p* > 0.05)] tests indicated that the ANOVA conditions of normality of residuals and homoscedasticity were satisfied.

It can be observed in Table 7 that there were significant main effects for the interaction between the alkaline treatment, red mud residue, and fiber length of F(2, 48) = 38.479, *p* < 0.001, for the treatment, F(1, 48) = 11.868, *p* < 0.001, for the red mud residue, F(2, 48) = 23.222, *p* < 0.001, and also for the fiber length, F(1, 48) = 20.892, *p* < 0.001. There were also significant interactions for X1:X2, which was F(2, 48) = 7.259, *p* < 0.001, X1:X3 of F(1, 48) = 16.1188, *p* < 0.001, and X2:X3 of F(2, 48) = 8.914, *p* < 0.001.

**Figure 8:**
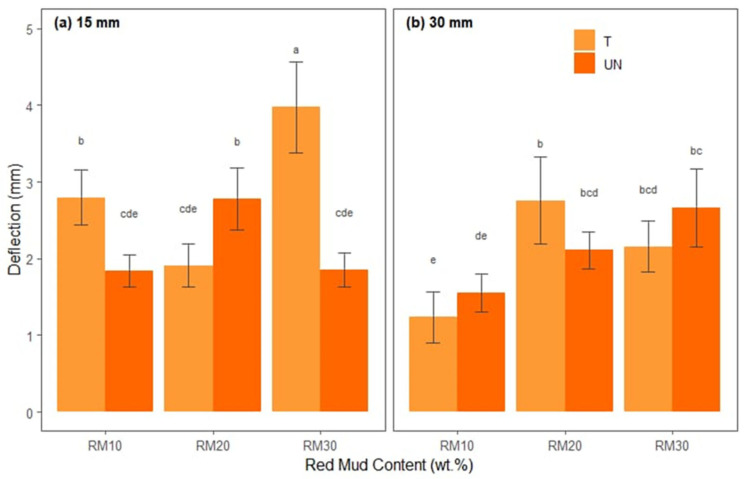
Statistical analysis of deflection for hybrid composites: (**a**) reinforced with 15 mm bamboo fibers; (**b**) reinforced with 30 mm bamboo fibers.

Figure 8 presents the average deflections of each treatment combination, separated into fiber lengths (Figure 8a,b) and proportions of red mud residue (*x*-axis) and differentiated by treated and untreated bamboo fibers.

In the deflection graphs (separated into 15 mm and 30 mm fibers with red mud contents of 10%, 20%, and 30%, in addition to treated and untreated fibers), a behavior almost opposite that of flexural strength is observed. In the case of 15 mm fibers (Figure 8a), some formulations with higher red mud content (30%), especially when untreated, exhibit the largest deflections (letters “a” or “b” in the legend), suggesting that although the material is more flexible, it can undergo more intense deformations before rupture. On the other hand, intermediate contents (20%) and treated fiber tend to reduce deflection, as the greater fiber–matrix adhesion and the moderate presence of mineral residue provide more rigidity and less deformation capacity.

For 30 mm fibers (Figure 8b), the pattern repeats itself in a similar way, but there are nuances linked to the greater length, which can increase the probability of failures due to delamination or scratching if the impregnation is not homogeneous. Still, the use of treated fiber tends to limit deflection, while untreated versions can reach higher values (letters “a” or “b”), reflecting less efficient adhesion and, therefore, greater possibility of deformation. Finally, Tukey’s test revealed that 15BTRMW30 (M = 3.97, SD = 0.59) had a mean deflection that was significantly greater than that of the other treatments.

The ANOVA results for the flexural modulus are presented in Table 8, which summarizes the main effects and interactions between the factors. The Shapiro–Wilk (W = 0.984, *p* > 0.05) and Levene [F(11.48) = 0.644, *p* > 0.05)] tests indicated that the ANOVA conditions of normality of residuals and homoscedasticity were also satisfied.

It is observed in Table 8 that there were significant main effects for fiber length, F(1, 48) = 111.90, *p* < 0.001, indicating that the mean flexural modulus behavior was significantly greater in the 15 mm bamboo fiber source (M = 20.074, SD = 5.46) than in the 30 mm bamboo fiber source (M = 15.580, SD = 3.287) for X2, F(2, 48) = 24.00, *p* < 0.001, and also for fiber length, F(1, 48) = 111.90, *p* < 0.001. There were also significant interactions for X1:X2, which was F(2, 48) = 8.22, *p* < 0.001, X1:X3, which was F(1, 48) = 12.43, *p* < 0.001, X2:X3, which was F(2, 48) = 27.95, *p* < 0.001, and X1:X2:X3, which was F(2, 48) = 24.29, *p* < 0.001.

**Figure 9:**
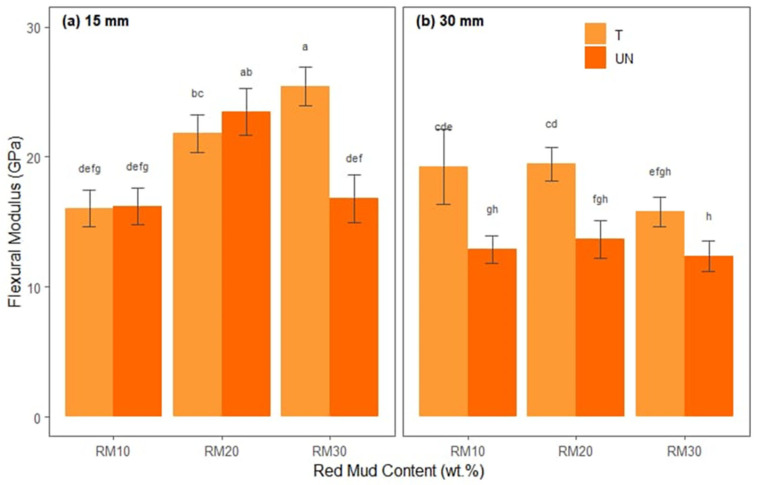
Statistical analysis of flexural modulus for hybrid composites: (**a**) reinforced with 15 mm bamboo fibers; (**b**) reinforced with 30 mm bamboo fibers.

Figure 9 shows the average flexural moduli of each treatment combination separated into fiber lengths (Figure 9a,b) and red mud waste proportions (*x*-axis) and differentiated by treated and untreated bamboo fibers.

In the flexural modulus graphs (separately for 15 mm and 30 mm fibers with red mud contents of 10%, 20%, and 30%, in addition to treated and untreated fibers), it can be seen that, in general, the chemical treatment of the fibers significantly increases the modulus, as it promotes better fiber–matrix adhesion and reduces interface failures. In the case of 15 mm fibers (Figure 9a), formulations with 20% or 30% red mud and treated fiber tend to achieve higher values (indicated by letters like “a” or “ab”), reflecting a stiffer composite. On the other hand, combinations with a lower red mud content (10%) or without chemical treatment present lower modules (letters “d”, “e”, “f”, etc.), suggesting that the reduced amount of mineral filler, combined with lower adhesion, results in lower rigidity.

For the 30 mm fibers (Figure 9b), the reinforcement effect of the treatment is still noticeable, but the greater extension of the fibers can introduce variations in dispersion and impregnation. Still, it is observed that the treated samples, especially with 20% or 30% red mud, tend to present higher values (letters like “bc” or “ab” compared to “gh” or “h” in the lower bars), indicating that the greater mineral fraction and the chemical adhesion between fiber and matrix increase the resistance to deformation. Samples without treatment or with a lower red mud content (10%) tend to exhibit lower modules, as the mineral fraction is insufficient to provide great rigidity and the lack of treatment reduces the efficiency of load transfer through the fibers. Finally, Tukey’s test revealed that 15BTRMW30 (M = 25.44, SD = 1.46) had a mean flexural modulus that was significantly higher than that of the other treatments.

#### 3.4.2. Statistical Analysis of Tensile Properties

The ANOVA results for tensile strength are presented in Table 9, which summarizes the main effects and interactions between the factors. The Shapiro–Wilk (W = 0.977, *p* > 0.05) and Levene [F(11.48) = 1.415, *p* > 0.05)] tests indicated that the ANOVA conditions of normality of residuals and homoscedasticity were satisfied.

It is observed in Table 9 that there were significant main effects for fiber length, F(1, 48) = 44.147, *p* < 0.001, indicating that the mean tensile strength was significantly higher in the 15 mm bamboo fiber source (M = 33.025, SD = 13.258) than in the 30 mm bamboo fiber source (M = 26.677, SD = 4.307) and also for the presence of red mud residue, F(2, 48) = 17.816, *p* < 0.001. However, the fiber treatment interaction, F(1, 48) = 0.158, *p* > 0.05, the interaction between X1 and X2, which was F(2, 48) = 2.178, *p* > 0.05, X1:X3 of F(1, 48) = 1.477, *p* > 0.05, X2:X3 of F(2, 48) = 2.248, *p* > 0.05, and X1:X2:X3, F(2, 48) = 2.920, *p* > 0.05, presented in Table 9, were not significant.

**Figure 10:**
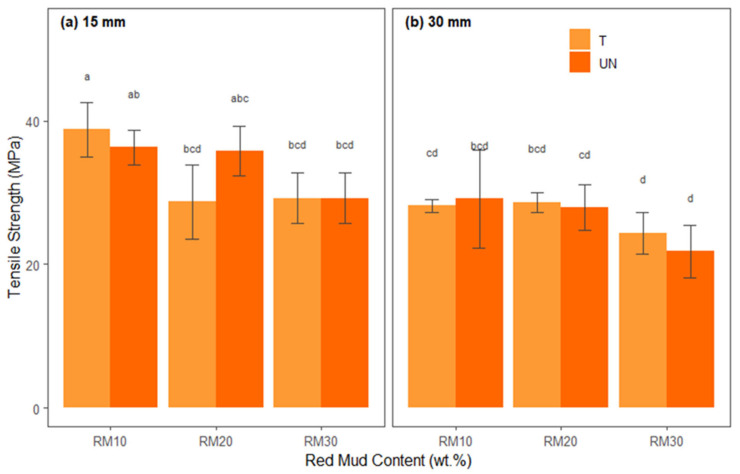
Statistical analysis of tensile strength for hybrid composites: (**a**) reinforced with 15 mm bamboo fibers; (**b**) reinforced with 30 mm bamboo fibers.

Figure 10 shows the average tensile strengths of each treatment combination separated into fiber lengths (Figure 10a,b) and proportions of red mud residue (*x*-axis) and differentiated by treated and untreated bamboo fibers.

The tensile strength graphs show that for 15 mm fibers (Figure 10a), the addition of 10% red mud and chemical treatment of the fibers tend to generate the highest breaking stresses (bars identified by the letters “a” or “ab”), suggesting that this residue content, combined with better fiber–matrix adhesion, provides the best performance. As the red mud content increases to 20% or 30%, a slight drop in resistance is observed, especially when there is no treatment of the fibers (bars with letters like “bcd” or “d”), which indicates that the excess mineral residue can introduce points of stress concentration or agglomerations, damaging the cohesion of the composite.

For the 30 mm fibers (Figure 10b), the difference between treated and untreated fibers remains evident, but, in general, the strength values are slightly lower than those for 15 mm. This may occur because the greater length of the fibers hinders homogeneous dispersion or generates localized flaws at the interface. Thus, even with treatment, formulations with 30% red mud usually present the lowest resistance (letters “c” or “d”), suggesting embrittlement due to the high mineral load or impregnation problems. The addition of 10% or 20% red mud to treated fibers maintains intermediate resistance (bars with letters “b” or “bc”).

The presence of different letters on the bars (a, b, c, etc.) confirms relevant statistical differences, reinforcing that the chemical treatment of the fibers and moderate levels of red mud (10% or 20%) result in better tensile strength values, while the increase to 30% and/or the absence of chemical treatment reduce the support capacity of the composite. Finally, Tukey’s test revealed that 15BTRMW10 (M = 38.81, SD = 3.84) tensile strength means were significantly higher than those of the other treatments.

The ANOVA results for strain are presented in Table 10, which summarizes the main effects and interactions between the factors. The Shapiro–Wilk (W = 0.977, *p* > 0.05) and Levene [F(11.48) = 1.060, *p* > 0.05)] tests indicated that the ANOVA conditions of normality of residuals and homoscedasticity were satisfied.

It can be seen in Table 10 that there were significant main effects for the interaction between alkaline treatment and fiber length F (1, 48) = 11.862, *p* < 0.001 and also for the interaction between alkaline treatment, red mud residue, and fiber length F (2, 48) = 8.774, *p* < 0.001. However, the fiber treatment interaction, F(1, 48) = 3.702, *p* > 0.05, the red mud residue interaction, F(2, 48) = 1.179, *p* > 0.05, the fiber length interaction, F(1, 48) = 2.652, *p* > 0.05, the interaction between X1 and X2, which was F(2, 48) = 3.730, *p* > 0.05, and for X2:X3, which was F(2, 48) = 2.496, *p* > 0.05, presented in Table 10, were not significant.

**Figure 11:**
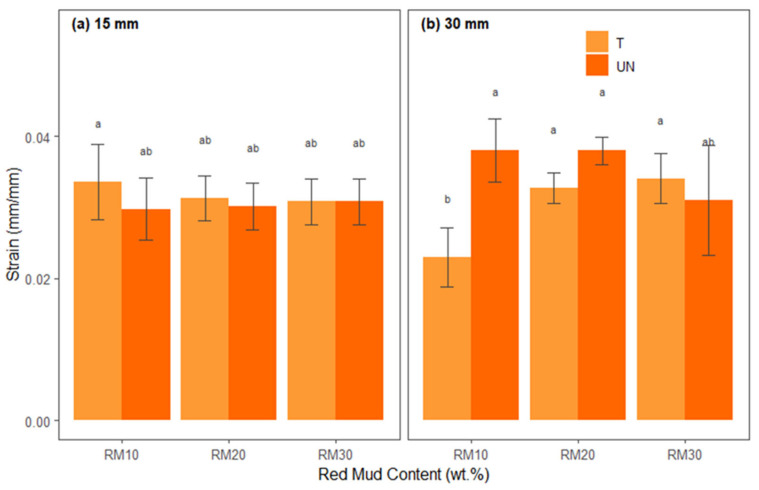
Statistical analysis of strain for hybrid composites: (**a**) reinforced with 15 mm bamboo fibers; (**b**) reinforced with 30 mm bamboo fibers.

Figure 11 presents the average deformations of each treatment combination separated into fiber lengths (Figure 11a,b) and proportions of red mud residue (*x*-axis) and differentiated by treated and untreated bamboo fibers.

When comparing the deformation graphs for 15 mm (a) and 30 mm (b) fibers, it can be seen that, in general, the values do not present large amplitudes but are still distributed in statistically distinct ranges, as indicated by the letters (a, b, ab, etc.). For 15 mm, some formulations with 10% red mud exhibit the greatest deformations (letter “a”), suggesting that the reduced mineral residue content and the shorter fiber length provide greater elongation capacity. Increasing the red mud content to 20% or 30%, especially in treated fibers, tends to slightly reduce deformation (bars with letters “ab” or “bcd”), as the more effective fiber–matrix adhesion and the greater fraction of mineral filler make the composite more rigid, reducing elongation before rupture.

In the case of 30 mm fibers, the variation in deformation follows a similar pattern, but with specific differences. In some combinations, the greater length may allow for microcracks or localized failures, increasing the average deformation (bars with letter “a”), while in others the presence of 30% red mud and chemical treatment generate additional rigidity and reduce ductility (letters “b” or “c”). The distinct letters above the bars confirm that these discrepancies are statistically significant; conditions with lower red mud content and/or untreated fibers can withstand larger deformations, while increasing waste content and treatment tend to reduce elongation, even while improving other mechanical parameters. Finally, Tukey’s test revealed that 30BURMW10 (M = 0.0380, SD = 0.0044) and 30BURMW20 (M = 0.0380, SD = 0.0020) had mean deformations that were significantly greater than those of the other treatments.

The ANOVA results for the elastic modulus are presented in Table 11, which summarizes the main effects and interactions between the factors. The Shapiro–Wilk (W = 0.978, *p* > 0.05) and Levene [F(11.48) = 1.700, *p* > 0.05)] tests indicated that the ANOVA conditions of normality of residuals and homoscedasticity were also satisfied.

It is observed in Table 11 that there were significant main effects for fiber length, F(1, 48) = 125.70, *p* < 0.001, indicating that the behavior of the mean elastic modulus was significantly higher in the 15 mm bamboo fiber source (M = 1.308, SD = 0.162) than in the 30 mm bamboo fiber source (M = 0.919, SD = 0.151), which was also significant for the red mud residue, F(2, 48) = 5.591, *p* < 0.001, as well as for X1:X2:X3, which was F(2, 48) = 5.825, *p* < 0.001. However, the fiber treatment interaction, F(1, 48) = 0.427, *p* > 0.05, the interaction between X1 and X2, which was F(2, 48) = 1.812, *p* > 0.05, the interaction between X1:X3, which was F(1, 48) = 1.982, *p* > 0.05, and X2:X3, which was F(2, 48) = 1.038, *p* > 0.05, presented in Table 11, were not significant.

**Figure 12:**
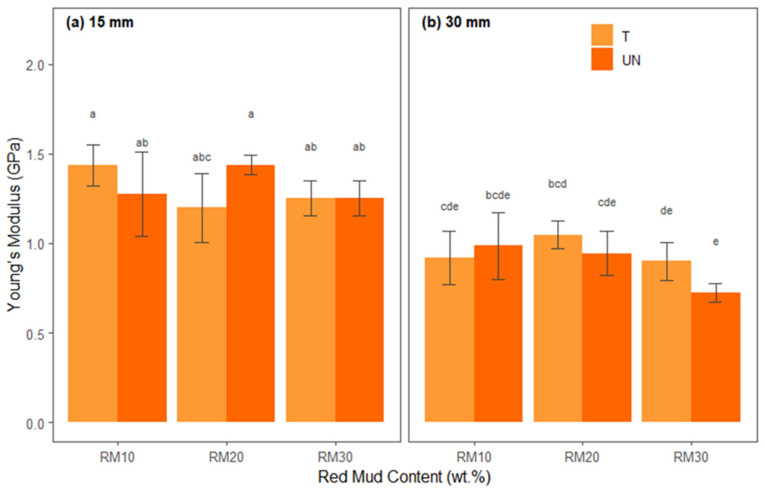
Statistical analysis of elasticity modulus for hybrid composites: (**a**) reinforced with 15 mm bamboo fibers; (**b**) reinforced with 30 mm bamboo fibers.

Figure 12 shows the average elastic moduli of each treatment combination, separated into fiber lengths (Figure 12a,b) and red mud waste proportions (*x*-axis) and differentiated by treated and untreated bamboo fibers.

In the elasticity modulus graphs, the bars corresponding to the 15 mm fibers Figure 12a show that combinations with chemical treatment (T) and moderate levels of red mud (10% or 20%) tend to achieve higher values (letters “a” or “ab”), suggesting good fiber–matrix adhesion and adequate reinforcement by the mineral filler. When the red mud content is increased to 30% or untreated fiber is used, the modulus tends to fall (bars with letters like “abc” or “b”), indicating less homogeneous dispersion or insufficient adhesion.

In the graph for 30 mm fibers Figure 12b, in general, slightly lower absolute values are observed, possibly due to difficulty in impregnation or a greater propensity for local failures in longer fibers. Still, the presence of chemical treatment and moderate residue content (20%) favors a gain in stiffness (bars with letters “bcd” or close to it), while combinations without treatment or with 30% red mud tend to present a lower modulus (letters like “e” or “cde”), reflecting greater heterogeneity and lower efficiency in load transfer.

The distinct letters (a, b, c, etc.) above the columns confirm that these differences are statistically significant, reinforcing the importance of controlling the residue content and fiber treatment to optimize composite stiffness. Finally, Tukey’s test revealed that 15BURMW20 (M = 1.436, SD = 0.055) and 15BTRMW10 (M = 1.435, SD = 0.114) had significantly higher elastic moduli than the other treatments.

### 3.5. Microstructural Analysis

**Figure 13:**
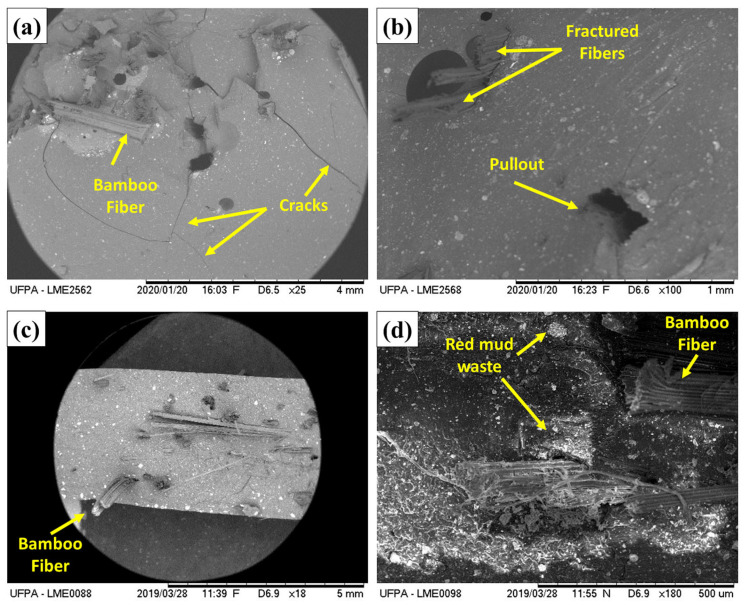
SEM images of the fracture surfaces of the composites following flexural testing. (**a**) 15BURMW10 (×25), (**b**) 30BURMW10 (×100), (**c**) 15BURMW20 (×18), and (**d**) 15BURMW30 (×180).

The SEM images of the broken surfaces of the analyzed composites following the flexural test are shown in Figure 13. They were utilized to examine the interfacial features and surface structure of the broken specimens concerning the observed mechanical performance.

In the micrographs, it is observed that the matrix presents cracks that propagate through the flexural region, indicating fracture of the resin when reaching its limit tension. In some areas, the bamboo fibers appear partially exposed or pulled out, suggesting incomplete adhesion at certain points, which facilitates fiber–matrix detachment. In other places, transverse fracture of fibers is noted, showing that, when well-anchored, the fibers resist until they break due to shear or traction in the region of greatest flexion. It is also possible to see micro voids or porosities, as well as faults around the red mud particles, where stress concentrations can occur that initiate cracks. These failure mechanisms—cracks in the matrix, fiber pull-out, fiber fracture, and detachment at the fiber–matrix interface, in addition to cracks in the region of mineral particles—are characteristic of composites in flexural conditions and explain the loss of structural integrity when the load exceeds the deformation capacity of the material.

**Figure 14:**
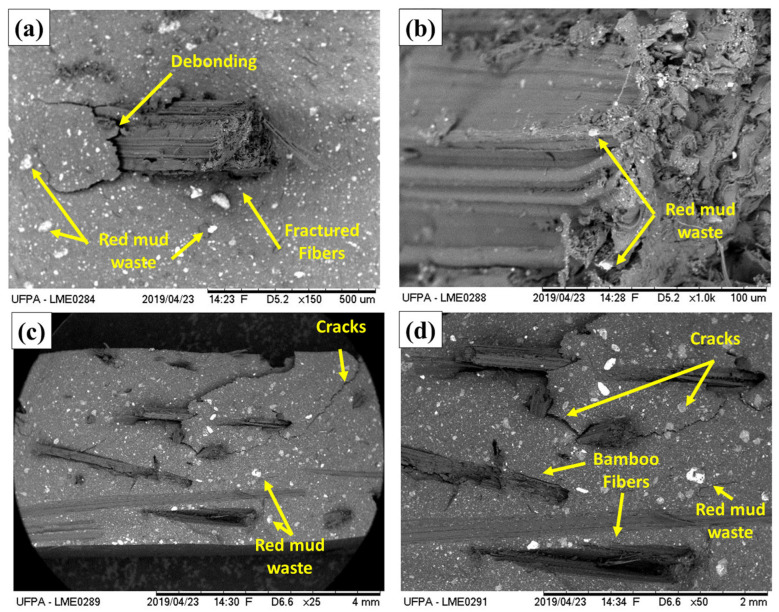
SEM images of the fracture surfaces of the composites following tensile testing. (**a**,**b**) 15BTRMW10 (×150, ×1000), (**c**,**d**) 15BTRMW20 (×25, ×50).

Figure 14 displays the fracture surfaces post tensile tests of hybrid composites reinforced with bamboo fiber and red mud waste.

From the micrographs, it is possible to identify several failure mechanisms typical of composites reinforced with natural fibers and mineral filler. Firstly, transversely fractured bamboo fibers are observed, showing that, at some points, the tensile stress exceeded the fiber’s resistance, leading to its rupture. The occurrence of “pull-out” (partial pulling out of fibers) is also noted, as some fibers appear detached or with gaps around them, indicating failures in the adhesion between the fiber and the matrix. Furthermore, there are regions of debonding at the fiber–matrix interface, where the contact between the bamboo and the resin appears to have broken before the fiber or matrix fractures, suggesting incomplete or compromised adhesion due to the presence of mineral residue or voids. Finally, small fragments and microcracks within the matrix and around the red mud grains show that the material may undergo crack propagation through the resin or around the particles, aggravating the overall rupture. Thus, the main failure mechanisms include fiber fracture, pull-out, delamination at the fiber–matrix interface, and microcracks in the matrix and the red mud particle region.

In general, the characterization of the internal morphology through Scanning Electron Microscopy (SEM) allowed for the qualitative verification of fiber dispersion, as well as the quality of the fiber–matrix interface. Through cross-sections and micrographs of the fracture surface, it was possible to identify whether the fibers are adequately embedded in the matrix or whether there are voids, detachments, agglomerates, or preferential orientation. Although SEM only provides localized two-dimensional information, its use is sufficient to infer the quality of the distribution and interfacial adhesion.

Bamboo fibers, due to their lignocellulosic nature, are composed mainly of cellulose, hemicellulose, and lignin, and they have a large amount of hydroxyl groups (–OH) in their structure. This makes them highly hydrophilic, promoting low chemical compatibility with the polyester matrix, which is hydrophobic. When used without surface treatment, the fibers tend to present low interfacial adhesion, moisture absorption, and, in some cases, partial exfoliation. This exfoliation manifests as separation of microfibrils, which may not be adequately impregnated by the matrix, creating regions of structural weakness.

These effects were observed in SEM analyses, where poorly adhered fibers are seen with a “clean” surface or detached from the matrix, suggesting failure through interfacial detachment. Furthermore, the presence of micro voids or filling failures can be attributed to the repulsion between the constituents and the poor wettability of the matrix over the fibrous surface.

To mitigate these effects, alkaline treatment with 5% NaOH for 2 h was adopted, a procedure that promotes the removal of hemicellulose and amorphous lignin, in addition to increasing the surface roughness of the fibers, which favors mechanical anchoring and chemical adhesion with the matrix. The mechanical results confirmed the effectiveness of this treatment, with the composites containing treated fibers presenting superior performance in both tensile and flexural strength, especially in formulations with higher red mud content.

## 4. Conclusions

This study presented the development of hybrid polyester matrix composites reinforced with bamboo fibers and red mud waste with the aim of creating sustainable and high-performance engineering materials. The results showed that the incorporation of red mud, in combination with treated and untreated fibers, significantly influenced the mechanical properties of the composites.

The 30BTRMW30 and 15BURMW20 composites demonstrated the best performances in flexural strength, with increases of up to 50.5% compared to pure polyester. For tensile strength, the 15BTRMW10 composite stood out, presenting a 68% increase in tensile strength compared to the reference sample. SEM micrographs revealed failure mechanisms typical of natural composites, such as fiber rupture, matrix cracking, and interfacial debonding. Statistical analysis validated the significance of the observed variations, confirming the influence of the formulation parameters.

These findings reinforce the potential of hybrid composites based on red mud waste and natural fibers as viable and environmentally responsible alternatives for applications in sectors that require moderate to high mechanical performance. As a future perspective, it is suggested to deepen the thermal, chemical, and durability characterization, in addition to application in functional prototypes to evaluate performance under real conditions of use.

## Figures and Tables

**Table 1:** Naming and structure of sample groups.

Fiber Length (mm)	Alkali-Treated 3 wt.%	Red Mud Waste (wt.%)	Sample Code
15		10	15BURMW10
15		20	15BURMW20
15		30	15BURMW30
30	Untreated	10	30BURMW10
30		20	30BURMW20
30		30	30BURMW30
15		10	15BTRMW10
15		20	15BTRMW20
15	Treated	30	15BTRMW30
30		10	30BTRMW10
30		20	30BTRMW20
30		30	30BTRMW30
-	Neat Polyester	-	NP

**Table 2:** Density of hybrid composites.

Sample	R.M.W. ^a^ (%)	F.L. ^b^ (mm)	A.T. ^c^ 3% wt.	Den. ^d^ (g/cm^3^)
NP ^e^	NA ^f^	NA ^f^	NA ^f^	1.26 ± 0.09
15BURMW10	10	15	Untreated bamboo fiber	1.24 ± 0.03
15BURMW20	20			1.30 ± 0.03
15BURMW30	30			1.41 ± 0.03
30BURMW10	10	30		1.51 ± 0.11
30BURMW20	20			1.39 ± 0.09
30BURMW30	30			1.42 ± 0.08
15BTRMW10	10	15	Treated bamboo fiber	1.32 ± 0.07
15BTRMW20	20			1.34 ± 0.09
15BTRMW30	30			1.42 ± 0.07
30BTRMW10	10	30		1.26 ± 0.08
30BTRMW20	20			1.37 ± 0.09
30BTRMW30	30			1.43 ± 0.09

^a^ Red mud waste; ^b^ fiber length; ^c^ alkali-treated; ^d^ density; ^e^ neat polyester; ^f^ not applicable.

**Table 3:** Flexural strength, deflection, and flexural modulus of hybrid composites.

Sample		R.M.W. ^a^ (%)	F.L. ^b^ (mm)	A.T. ^c^ 3% wt.	F.S. ^d^ (MPa)	Def ^e^ (mm)	F.M. ^f^ (GPa)
NP ^g^		NA ^h^	NA ^h^	NA ^h^	82.86 ± 3.19	1.25 ± 0.38	13.80 ± 1.22
S.V. ^i^	→	X1	X2	X3	Y1	Y2	Y3
15BURMW10		10	15	Untreated bamboo fiber	104.53 ± 4.07	1.84 ± 0.21	16.07 ± 1.60
15BURMW20		20			115.51 ± 5.96	2.78 ± 0.40	23.48 ± 1.79
15BURMW30		30			83.79 ± 3.23	1.86 ± 0.22	16.80 ± 1.82
30BURMW10		10	30		86.68 ± 3.39	1.55 ± 0.25	12.91 ± 1.05
30BURMW20		20			88.26 ± 3.73	2.11 ± 0.24	13.67 ± 1.47
30BURMW30		30			84.72 ± 4.96	2.66 ± 0.51	12.38 ± 1.18
15BTRMW10		10	15	Treated bamboo fiber	100.44 ± 4.64	2.80 ± 0.36	16.04 ± 1.40
15BTRMW20		20			113.93 ± 3.36	1.91 ± 0.29	21.81 ± 1.47
15BTRMW30		30			97.65 ± 3.59	3.97 ± 0.59	25.44 ± 1.46
30BTRMW10		10	30		103.70 ± 5.60	1.24 ± 0.33	19.26 ± 2.93
30BTRMW20		20			111.97 ± 5.48	2.76 ± 0.57	19.47 ± 1.28
30BTRMW30		30			124.71 ± 4.35	2.16 ± 0.33	15.79 ± 1.13

^a^ Red mud waste; ^b^ fiber length; ^c^ alkali-treated; ^d^ flexural strength; ^e^ deflection; ^f^ flexural modulus; ^g^ neat polyester; ^h^ not applicable; ^i^ statistical variables.

**Table 4:** Tensile strength, total strain, and Young’s modulus of hybrid composites.

Sample	R.M.W. ^a^ (%)	F.L. ^b^ (mm)	A.T. ^c^ 3% wt.	T.S. ^d^ (MPa)	To.S ^e^ (mm/mm)	Y.M. ^f^ (GPa)
NP ^g^	NA ^h^	NA ^h^	NA ^h^	23.11 ± 4.04	0.0316 ± 0.0037	0.924 ± 0.096
S.V. ^i^	X1	X2	X3	Y1	Y2	Y3
15BURMW10	10	15	Untreated bamboo fiber	36.33 ± 2.44	0.0351 ± 0.0043	1.275 ± 0.233
15BURMW20	20			35.82 ± 3.40	0.0301 ± 0.0033	1.436 ± 0.055
15BURMW30	30			29.23 ± 3.49	0.0308 ± 0.0032	1.253 ± 0.096
30BURMW10	10	30		29.14 ± 6.82	0.0380 ± 0.0044	0.985 ± 0.184
30BURMW20	20			27.93 ± 3.12	0.0380 ± 0.0020	0.943 ± 0.123
30BURMW30	30			21.79 ± 3.69	0.0310 ± 0.0078	0.723 ± 0.050
15BTRMW10	10	15	Treated bamboo fiber	38.81 ± 3.84	0.0335 ± 0.0053	1.435 ± 0.114
15BTRMW20	20			28.72 ± 5.18	0.0312 ± 0.0031	1.197 ± 0.190
15BTRMW30	30			29.23 ± 3.49	0.0308 ± 0.0032	1.253 ± 0.096
30BTRMW10	10	30		28.15 ± 0.84	0.0230 ± 0.0041	0.921 ± 0.148
30BTRMW20	20			28.66 ± 1.40	0.0328 ± 0.0021	1.046 ± 0.076
30BTRMW30	30			24.39 ± 2.91	0.0340 ± 0.0035	0.898 ± 0.105

^a^ Red mud waste; ^b^ fiber length; ^c^ alkali-treated; ^d^ tensile strength; ^e^ total strain; ^f^ Young’s modulus; ^g^ neat polyester; ^h^ not applicable; ^i^ statistical variables.

**Table 5:** Comparison of the results found in the present work with those in the literature.

Sample	Alkali-Treated	Red Mud Waste (wt.%)	Flexural Strength (MPa)	Tensile Strength (MPa)	Reference
30BTRMW30	Treated	30	124.71 ± 4.35	24.39 ± 2.91	This study
15BURMW20	Untreated	20	115.51 ± 5.96	35.82 ± 3.40	
15BURMW10	Untreated	10	104.53 ± 4.07	36.33 ± 2.44	
15BTRMW10	Treated	10	100.44 ± 4.64	38.81 ± 3.84	
RW-2	Untreated	40	50.19	18.93	[14]
PMC-PP-10	Untreated	10	55.4	30.0	[15]
EBR-4	Untreated	15	28.1	12.5	[20]
UEUBRMC_0_	Untreated	0	135.13	165.1	[28]
UETBRMC_0_	Treated	0	120.12	195.2	
UETBRMC_5_	Treated	5	130.3	205.2	
UETBRMC_10_	Treated	10	165.2	255.1	
UETBRMC_15_	Treated	15	215.3	149.2	

**Table 6:** Analysis of variance for flexural strength (MPa).

Sources	df	SQ	QM	F	*p* (>F)
X_1_	1	4967	4967	249.89	0
X_2_	2	2703	1352	67.99	0
X_3_	1	543	543	27.29	0
X_1_:X_2_	2	1114	557	28.03	0
X_1_:X_3_	1	1138	1138	57.26	0
X_2_:X_3_	2	3763	1882	94.66	0
X_1_:X_2_:X_3_	2	304	152	7.65	0
Residuals	48	954	20		

**Table 7:** Analysis of variance for deflection (mm).

Sources	df	SQ	QM	F	*p* (>F)
X_1_	1	1.720	1.720	11.868	0
X_2_	2	6.732	3.366	23.222	0
X_3_	1	3.029	3.029	20.892	0
X_1_:X_2_	2	2.105	1.105	7.259	0
X_1_:X_3_	1	2.336	2.336	16.118	0
X_2_:X_3_	2	2.584	1.292	8.914	0
X_1_:X_2_:X_3_	2	11.156	5.578	38.479	0
Residuals	48	6.958	0.145		

**Table 8:** Analysis of variance for flexural modulus (GPa).

Sources	df	SQ	QM	F	*p* (>F)
X_1_	1	208.51	208.51	80.94	0
X_2_	2	123.67	61.83	24.00	0
X_3_	1	288.25	288.25	111.90	0
X_1_:X_2_	2	42.35	21.17	8.22	0
X_1_:X_3_	1	32.02	32.02	12.43	0
X_2_:X_3_	2	144.00	72.00	27.95	0
X_1_:X_2_:X_3_	2	125.12	62.56	24.29	0
Residuals	48	123.64	2.58		

**Table 9:** Analysis of variance for tensile strength (MPa).

Sources	df	SQ	QM	F	*p* (>F)
X_1_	1	2.20	2.20	0.158	0.692
X_2_	2	487.90	244.0	17.816	0
X_3_	1	604.5	604.5	44.147	0
X_1_:X_2_	2	59.60	29.80	2.178	0.124
X_1_:X_3_	1	20.20	20.20	1.477	0.230
X_2_:X_3_	2	61.60	30.80	2.248	0.116
X_1_:X_2_:X_3_	2	80.00	40.00	2.920	0.063
Residuals	48	657.30	13.70		

**Table 10:** Analysis of variance for strain (mm/mm).

Sources	df	SQ	QM	F	*p* (>F)
X_1_	1	6.324 × 10^−5^	6.324 × 10^−5^	3.702	0.060
X_2_	2	4.030 × 10^−5^	2.014 × 10^−5^	1.179	0.316
X_3_	1	4.530 × 10^−5^	4.530 × 10^−5^	2.652	0.109
X_1_:X_2_	2	12.740 × 10^−5^	6.372 × 10^−5^	3.730	0.051
X_1_:X_3_	1	2.026 × 10^−4^	2.026 × 10^−4^	11.862	0
X_2_:X_3_	2	8.530 × 10^−5^	4.264 × 10^−5^	2.496	0.093
X_1_:X_2_:X_3_	2	2.998 × 10^−4^	1.499 × 10^−4^	8.774	0
Residuals	48	82.00 × 10^−5^	1.708 × 10^−5^		

**Table 11:** Analysis of variance for modulus of elasticity (GPa).

Sources	df	SQ	QM	F	*p* (>F)
X_1_	1	0.007	0.007	0.427	0.516
X_2_	2	0.201	0.100	5.591	0
X_3_	1	2.269	2.269	125.70	0
X_1_:X_2_	2	0.065	0.032	1.812	0.174
X_1_:X_3_	1	0.035	0.035	1.982	0.165
X_2_:X_3_	2	0.037	0.018	1.038	0.362
X_1_:X_2_:X_3_	2	0.210	0.105	5.825	0
Residuals	48	0.866	0.018		

## Data Availability

The original contributions presented in the study are included in the article; further inquiries can be directed to the corresponding author.
